# Concurrent and Predictive Criterion Validity of a Puppy Behaviour Questionnaire for Predicting Training Outcome in Juvenile Guide Dogs

**DOI:** 10.3390/ani10122382

**Published:** 2020-12-11

**Authors:** Rebecca L. Hunt, Gary C. W. England, Lucy Asher, Helen Whiteside, Naomi D. Harvey

**Affiliations:** 1 Guide Dogs National Breeding Centre, Banbury Road, Bishops Tachbrook, Warwickshire CV33 9WF, UK; Helen.VaterlawsWhiteside@guidedogs.org.uk; 2 School of Veterinary Science & Medicine, University of Nottingham, Sutton Bonington Campus, Leicestershire LE12 5RD, UK; gary.england@nottingham.ac.uk (G.C.W.E.); naomi.harvey@nottingham.ac.uk (N.D.H.); 3 Centre for Behaviour and Evolution, School of Natural and Environmental Sciences, Newcastle University, Agriculture Building, Newcastle NE1 7RU, UK; lucy.asher@newcastle.ac.uk

**Keywords:** guide dog, assistance dog, behaviour, prediction, personality, test

## Abstract

**Simple Summary:**

The ability to predict later success in guide dog training can be of great benefit to assistance dog providers, such as those providing guide dogs, to ensure maximum resource and production efficiency and to maintain high welfare standards. This study evaluated the predictive capabilities of a behaviour questionnaire (the refined puppy walker questionnaire, r-PWQ) completed by volunteer carers of puppies (puppy walkers) for dogs aged eight months of age. The r-PWQ includes traits such as “Distractibility” and “Excitability”, which are common withdrawal reasons for many guide dogs. The predictive validity of the r-PWQ was compared to a well-known behaviour questionnaire, (Canine Behavioral Assessment and Research Questionnaire—C-BARQ). The results show that the r-PWQ can be used to predict guide dog outcome and may be better suited to guide dog populations than the C-BARQ.

**Abstract:**

Working dog organisations regularly assess the behaviour of puppies to monitor progression. Here, we tested the predictive validity (for predicting success in guide dog training) of a shortened version of a previously developed juvenile dog behaviour questionnaire (the refined puppy walker questionnaire, r-PWQ) and compared it with the Canine Behavioral Assessment and Research Questionnaire (C-BARQ). The r-PWQ is used by Guide Dogs UK, whereas the C-BARQ was designed for pet dogs and is used by some other guide dog schools internationally. A cohort of dogs aged eight months (n = 359) were scored concurrently on the r-PWQ and C-BARQ. Analogous traits between the questionnaires were evaluated for internal consistency and association with training outcome and compared for concurrent validity. The r-PWQ was associated with training outcome for five scales (r-Excitability, Trainability, Animal Chase, r-Attachment and attention seeking and Distractibility) and the C-BARQ for two scales (Excitability and Separation-related behaviour). There were significant correlations between analogous C-BARQ and r-PWQ trait scores (*p* < 0.001) except for Separation-related behaviour and questionnaire scales had similar internal consistencies. The r-PWQ may be more suitable to use with guide dog schools. However, due to the correlation between analogous scales (except for “Distractibility”) some scales could be substituted for one another when reviewing the behaviour of dogs between guide dog schools using different questionnaires.

## 1. Introduction

Undesirable behaviour is reported to be the most frequent reason for withdrawal of dogs from guide dog training programmes [1,2,3,4]. Therefore, it is essential to monitor behaviour and identify problems as early as possible. Identification of behaviours associated with withdrawal is of great benefit to guide dog schools in terms of minimising costs, maintaining production efficiency and high welfare standards. Costs and resources can be saved if dogs that are unlikely to succeed as guide dogs can be recognised and removed from the programme before entering formal training. With fewer dogs in the training programme, staff have greater time to spend with each dog and therefore increase the chance of success for training dogs. The welfare of dogs is also improved by rehoming or repurposing dogs earlier that are not suitable for the guiding role.

With ever-increasing international collaborations, particularly across guide dog breeding programs, there would be many advantages of schools using the same methods of behaviour data collection. A more consistent international approach to behaviour data collection would enable guide dog schools to incorporate and utilise data on dogs transferred between schools (e.g., movement and selection of breeding stock or semen). By using consistent data collection tools, estimated breeding values for behaviour could be more easily shared across populations, which could inform breeding decisions and provide information on the offspring of breeding stock that have been transferred internationally. Such processes would provide greater scope for collaborative research projects and population data could be combined across schools to enable more effective and efficient future behavioural genomic analysis.

Many guide dog schools use questionnaires or observational puppy assessments to measure the behaviour of dogs during puppyhood and before being placed into training [5,6,7,8]. Several guide dog schools use questionnaires completed by volunteers who raise puppies, in a similar manner to the refined Puppy Walker Questionnaire (r-PWQ) [5,9]. Guide Dogs UK currently uses the r-PWQ, which is a refined version of the Puppy Walker Questionnaire (PWQ) [8], to obtain behavioural information on puppies. The r-PWQ was designed specifically for the guide dog population and comprises a series of 39 questions that form nine scales which are considered important to guide dog populations. Of the nine r-PWQ scales, three have been refined (r-Separation-related behaviour; r-Excitability; r-Attachment and attention seeking), five scales remain the same as in the PWQ (General Anxiety; Trainability; Body Sensitivity; Energy; Distractibility) and there is an additional scale named Animal Chase. Some other guide dog schools are using an adapted version of the Canine Behavioral Assessment and Research Questionnaire (C-BARQ) which was designed for the pet dog population [10,11]. The C-BARQ is designed to provide information on dog behaviour problems by measuring 14 traits (Stranger-directed aggression; Owner-directed aggression; Dog-directed aggression; Dog rivalry; Stranger-directed fear; Non-social fear; Dog-directed fear; Separation-related behaviour; Attachment and attention-seeking; Trainability; Chasing; Excitability; Touch sensitivity; Energy level) [10]. The C-BARQ database contains over 50,000 dog behaviour records and has been collecting data since 2005 [12].

As the C-BARQ was designed for a pet dog population, it could be argued that the questionnaire has limitations when applied to a working or guide dog population, however some results have been linked to success of guide dogs in the USA [11]. The C-BARQ does not include questions to provide trait scores for distraction or “distractibility”, a trait which has been reported as a common reason for withdrawal in guide dog programmes [1,2,4,5,13]. Additionally, it is possible that some questions may not be as relevant to guide dog populations [5]. While aggression, along with distraction and fear, have been suggested as important reasons for withdrawal from guide dog programmes, within guide dog breeding programmes dogs are selected to be non-aggressive and guide dog populations report lower numbers and levels of aggression than pet dog populations [14,15]. Due to the potentially less intense and less frequent behavioural displays of aggression shown by guide dogs, the C-BARQ scale may not be appropriate for capturing data on aggressive displays for guide dogs.

The aim of this study was to test the predictive ability of the r-PWQ in an independent cohort of juvenile guide dogs similar to those with which it was initially created. An additional aim was to directly compare the reliability and predictive validity of the C-BARQ and r-PWQ traits to determine if one would be more appropriate for use in guide dog populations. Results for the r-PWQ Distractibility trait (a scale for which there is no C-BARQ analogue) were evaluated to examine the effectiveness of this novel measurement as a reliable and predictable measure for guide dog populations.

## 2. Materials and Methods

### 2.1. Participants

A cohort of dogs and their puppy walkers (volunteers who live with and care for guide dog puppies) formed the basis of this study. Guide Dogs UK puppy walkers for dogs that reached eight months of age between April and October 2017 were contacted via email (n = 405) and asked to voluntarily complete the online questionnaire. The survey was further distributed to a group of puppy walkers from Guiding Eyes USA for puppies that turned eight months of age during the same period (the survey was distributed by Guiding Eyes USA so that no personal data was shared). Questionnaires were sent when a puppy turned 226 days old and remained open for four weeks until 254 days old. In total, 359 dogs (198M/161F) had completed questionnaire responses (321 from Guide Dogs UK and 38 from Guiding Eyes USA). The dogs comprised eight breeds or crossbreeds (German Shepherd Dog, 17; Golden Retriever, 49; Golden Retriever _Sire_ × German Shepherd Dog _Dam_, 5; Golden Retriever _Sire_ × Golden Retriever crossbreed _Dam_, 42; Golden Retriever _Sire_ × Labrador _Dam_, 41; Labrador, 159; Labrador _Sire_ × Golden Retriever _Dam_, 17; Labrador _Sire_ × Golden Retriever crossbreed _Dam_, 29). The dogs were a mean age of 7.80 months (±7 days S.D.) at questionnaire completion. Dogs entered formal guide dog training between 0.96 and 1.76 years of age (mean age for entry into training was 1.14 ± 0.11 years of age). Guide Dog training staff certified a dog as suitable to be a guide dog based on the organisations’ criteria. If deemed suitable for the guiding role, dogs were matched with clients and further training undertaken in order to qualify the dog and owner as a guide dog partnership. Following completion of this training, dogs were certified as ‘qualified’. Dogs deemed not suitable for the guiding role by qualified training staff were withdrawn and reasons for the withdrawal given. Dogs qualified between 1.46 and 2.76 years of age (mean qualification age was 1.84 ± 0.21 years of age). Information on whether a dog had qualified or was withdrawn was collected from Guide Dogs’ electronic database.

### 2.2. The Questionnaires

For data collection for this study, a single questionnaire was produced in SurveyGizmo (www.surveygizmo.eu) that contained all questions from the 100-item version of C-BARQ [9] and the 39 items from the r-PWQ (Table 1). A total of 140 questions were asked in randomised order (“Is self-controlled and calm” appeared in both r-Excitability and Trainability for the r-PWQ).

#### 2.2.1. r-PWQ

The r-PWQ contained 39 items in nine scales: r-Separation-related behaviour; r-Excitability; r-Attachment and attention seeking; Energy Level; General Anxiety; Animal Chase; Body Sensitivity; Distractibility and Trainability (Table 1). The r-PWQ was refined from the original PWQ (see Supplementary Materials, Refining the PWQ method) in a population of 276 dogs (Table S1). The PWQ contained 61 questions and 10 scales (all questions and scales can be found in Table S2). Animal Chase was a new scale which contained two questions originating from the C-BARQ scale Chasing (“Chases birds or squirrels (or would like to)” and “Chases cats (or would like to)”) and three scales had some items removed due to lack of individual associations with training outcome, or lack of reliability (see Table S2 for individual results for each item and details on how the PWQ was refined into the r-PWQ). In the r-PWQ, these scales were referred to as r-Attachment and attention seeking (r-AAS), r-Separation-related behaviour (r-SRB) and r-Excitability to indicate that they have been refined as compared to their original form in the original PWQ. r-PWQ questions were answered using a visual analogue scale from 0 to 100, where 0 = “Never” and 100 = “Almost Always” and the question was left blank if not applicable [8]. To replicate the C-BARQ methodology, when 25% or more of the trait questions were left blank the trait score was not calculated for that dog and the data recorded as missing [9]. Trait scores were calculated as the mean of individual question scores within each trait, with negatively oriented scores reversed before creating the mean score.

#### 2.2.2. C-BARQ

C-BARQ questions were answered using a five-point frequency scale (0 = Never; 1 = Seldom, 2 = Sometimes, 3 = Usually, 4 = Always) or a five-point severity scale (0 = no sign of the behaviour to 4 = a severe form of the behaviour) according to the C-BARQ 100 scoring criteria, with the exception of one question (“Dog acts aggressively towards cats, squirrels or other small animals entering your garden”) where due to an oversight, a frequency scale was used instead of a severity scale. An option for “Not observed/not applicable” was available for C-BARQ questions. Questions were grouped as previously described [10], producing 14 trait scores (Stranger-directed aggression; Owner-directed aggression; Dog-directed aggression; Dog rivalry; Stranger-directed fear; Non-social fear; Dog-directed fear; Separation-related behaviour; Attachment and attention-seeking; Trainability; Chasing; Excitability; Touch sensitivity; Energy level). When 25% or more of the trait questions were left blank or selected as “Not applicable”, the trait score was not calculated for that dog and the data recorded as missing [9]. Trait scores were calculated as the mean of individual question responses, with negatively oriented scores reversed before creating the mean score.

### 2.3. Score Comparisons

There were eight C-BARQ and r-PWQ traits compared during the study (Table 2). The Distractibility trait from the r-PWQ could not be compared to any C-BARQ trait but was included in all other analyses. The frequency and distribution of scores were checked before any comparisons were made. The percentage of dogs with completed trait scores was determined, alongside the minimum score, maximum score, mean, median and standard deviation for each trait. Differences between the two guide dog populations were checked and scores were largely similar supporting pooling of the data from the two populations (see Supplementary Materials, Comparison between Guide Dog populations). However, the scores for Body Sensitivity and Attachment and attention seeking traits in both the r-PWQ and C-BARQ did differ somewhat between populations. Internal reliability of C-BARQ and r-PWQ scales was assessed using Cronbach’s alpha. A histogram for responses for each trait was then used to examine distribution. For direct comparisons between C-BARQ and r-PWQ traits, Spearman’s Rank correlation coefficients were used. C-BARQ trait scores were first transferred to a C-BARQ 100 scale (by dividing them by 4 and multiplying by 100). Following this, Z-scores were calculated for all traits to determine a dog’s rank within the population as follows:

Z-score = (trait score − mean of population)/standard deviation of population.(1)

A univariate analysis of variance was used to determine whether breed and sex should be controlled for within the “population” section of the Z-score calculations. When trait scores were significantly impacted by breed or sex, these were controlled for within Z-scores by calculating separate means and standard deviations for breeds or sex. When trait scores were significantly impacted by both breed and sex (n = 2), the factor with the strongest association was controlled for.

### 2.4. Predictive Ability

For the prediction element of this study, only data from Guide Dogs UK dogs (n = 261) that had qualified as a guide dog (n = 171) or been withdrawn permanently for behavioral reasons (n = 90) were included in the analysis. Dogs that were selected as breeding stock and therefore did not enter formal guide dog training (n = 33), were withdrawn for health reasons (n = 24), or that had not reached outcome at the time of analysis (n = 3) were excluded from this section of the analysis. A binary logistic generalized model was used for each trait to determine whether the C-BARQ or r-PWQ Z-scores were linked to outcome as a guide dog. All results were considered significant when *p* < 0.05.

## 3. Results

### 3.1. Trait Score Comparisons

A total of 4.5% of the C-BARQ trait scores (from the 44 questions relating to the eight comparable C-BARQ traits) were left blank or “Not Applicable”, compared to just 1.4% of r-PWQ questions. Individual dogs had between 77.4% and 100.0% of completed trait scores due to questions left blank or selected as “Not Applicable”. The internal consistency for C-BARQ and r-PWQ trait scores ranged from 0.51 (C-BARQ Trainability) to 0.88 (r-PWQ Energy) (see Table 3). Four trait scores had a higher Cronbach’s alpha for the C-BARQ (Excitability, Non-social fear, Chasing and Attachment and attention-seeking) and four trait scores were higher for the r-PWQ (r-Separation-related behaviour, Trainability, Body Sensitivity and Energy). Three trait scores had a lower than acceptable Cronbach’s alpha of less than 0.60 (Separation-related behaviour on the C-BARQ and Trainability on both questionnaires), but the internal consistency was no lower than 0.50 (Table 3).

There was an impact of breed and sex on selected trait scores (Table 4). The comparable trait scores of C-BARQ Non-social fear and r-PWQ General Anxiety were affected by both breed and sex however breed was controlled for due to the higher impact. Similarly, breed needed to be controlled for in both C-BARQ Chasing and r-PWQ Animal Chase trait scores. No models controlled for the effects of sex. The effects of breed were controlled for by creating trait Z-scores using the mean and standard deviations for breeds when the effect was significant.

When comparing the correlation between C-BARQ and r-PWQ trait scores, there was no significant correlation for the Separation-related behaviour scores (Table 5). All other trait scores were moderately to highly correlated (correlation coefficients ranged from 0.42 to 0.78), with Chase scores having the highest correlation.

### 3.2. Predictive Ability

A total of 261 dogs were included in the qualification prediction analysis; 171 dogs (65.5%) qualified as guide dogs and 90 (34.5%) were withdrawn for behavioural reasons. There were two C-BARQ trait scores that were linked to outcome as a guide dog compared to five r-PWQ trait scores (Table 6). The confidence interval was greater for all C-BARQ trait scores when compared to the r-PWQ except for Fear/anxiety and Trainability. The Wald Chi-square was higher for the predictive r-PWQ trait scores (r-Excitability, Animal Chase, Trainability, r-Attachment and attention seeking) when compared to the correlated C-BARQ trait scores.

## 4. Discussion

Behaviour is the most common withdrawal reason for dogs in guide dog schools [9]. It has been reported that 77.3% of dogs withdrawn in Australia [1], 65.5% in the USA [2] and 69.5% in Japan [3] were for behavioural reasons. In Guide Dogs UK, approximately 75% of dogs that do not qualify as guide dogs are withdrawn for behavioural reasons [13]. It is worth noting that guide dog schools may measure success using slightly different methods and for different time periods dependent on the guide dog school’s processes and measures. However, if behaviours associated with withdrawal can be identified during the juvenile period, schools could intervene earlier and potentially resolve a behavioural issue or withdraw a dog before embarking on a training programme, resulting in improved efficiency. Furthermore, if there were consistency in behaviour measurements between guide dog schools, international collaborations, movement of breeding stock, monitoring of population demographics and collaborative research studies could be improved. This study found the r-PWQ to be predictive of guide dog success for five traits, compared with two of the eight analogous C-BARQ traits (there was no comparable C-BARQ trait for r-PWQ Distraction), suggesting the questionnaire could support working dog populations to aid decision making around suitability for a working role.

A common method for collecting behaviour data in a large and geographically wide-spread population is through questionnaires. Owner-reported behaviour questionnaires have been used in numerous studies, with the most commonly reported questionnaire being the C-BARQ [10]. The C-BARQ has been widely used to successfully record behaviour amongst different dog populations, including shelter, working and pet dogs [16,17,18,19,20], and amongst guide dog schools [2,9]. Some studies however have suggested that the C-BARQ may be unsuitable for certain dog populations. Foyer et al. [19] reported the predictive power of C-BARQ alone to be low for assessing the suitability of German Shepherd Dogs in becoming military dogs. Similarly, this study found the predictive power of the C-BARQ to be low for juvenile guide dog puppies at eight months, with only two trait scores significantly associated with guide dog success (Separation-related behaviour and Excitability). Both the C-BARQ and r-PWQ have previously reported acceptable inter-rater reliability [8,15]. The r-Separation-related behaviour trait scores in the C-BARQ were predictive of guide dog success, whereas they were not in the r-PWQ. The r-PWQ scale is six questions (five of which originated from the C-BARQ) whilst the C-BARQ equivalent score contains eight questions. Cronbach’s alphas for internal reliability were similar between the separation scores (0.56 C-BARQ, 0.60 r-PWQ), however there was no significant correlation between the two scores (*p* = 0.193). Results indicate that the r- Separation-related behaviour score could perhaps be improved if the C-BARQ Separation-related behaviour questions that were removed during refinement were added back into the r-PWQ. There are also some differences in the wording of questions between the C-BARQ and r-PWQ comparable Separation scores, which could further add to the differences found in predictive ability. The r-PWQ Distractibility score showed the highest predictive power of all scores, a trait which is novel when compared to C-BARQ. Batt et al. [5] similarly reported novel questions to be more significant for predicting guide dog success when compared to standard C-BARQ questions.

Distraction is a common behavioural reason for withdrawal from guide dog training programmes [1,2]. Throughout this study ‘Distraction’ was defined as attraction to items in the environment. The r-PWQ Distraction trait score showed the strongest association to future guide dog success, suggesting the trait may have the greatest predictive power at eight months of age. There is a lack of distractibility questions within the C-BARQ, which is of considerable importance to guide dog organisations [3,4,5,6]. Batt et al. [5] identified the need for additional questions to be asked to puppy raisers alongside C-BARQ questions in order to measure dog distraction for guide dogs in Australia. Arata et al. [3] reported distraction as the most important trait to measure for predicting guide dog qualification; dogs with lower distraction scores were significantly more likely to qualify, and when using distraction scores to predict later success 80.6% of dogs with scores under a set threshold qualified as guide dogs. Similarly, Kobayashi et al. [6] reported that distraction factor points measured at five months of age were significantly lower in guide dogs that qualified when compared to dogs that did not. The current study suggests that distraction questions are predictive of guide dog success at eight months of age and individual questions from the Distractibility trait score show predictability at five, eight and 12 months (see Table S2). The creators of the C-BARQ acknowledge that 13.6% of dogs rejected from guide dog training at the Seeing Eye (USA) were for distraction [2] and it is recommended that a new set of questions should be added to the questionnaire to measure distraction. Findings from a puppy test indicated that distraction behaviours observed in guide dog puppies at five and eight months of age contributed heavily to predictions of training outcome [21], which add further support to the need to measure this trait. There are arguably correlations between Animal chase and Distractibility traits, however for this guide dog population we found differences between the predictive abilities and impact of breed on the two r-PWQ traits, and internal reliability testing for Distractibility was best without the Animal chase questions included. Animal chase was significantly impacted by breed and Distractibility had a stronger predictive capability. As a result, the two trait scores remained separate in this study. Due to the importance within guide dog programmes, it is recommended that a behaviour questionnaire for potential guide dogs should incorporate an assessment of distractibility [3,4], therefore the r-PWQ may be a more appropriate option for juvenile guide dogs than the C-BARQ. There is the potential to explore the use of r-PWQ for other assistance or working dog populations.

Within working dog organisations, it is common for volunteers to raise puppies in the first year of life [2,6,8,19,22]. Information provided by these volunteers has been reported to predict working dog success for guide [2,5,6] and other working dogs [19]. Similarly, this study found that puppy walkers were able to report behavioural data that could accurately predict guide dog outcome. The information collected can also aid volunteers and staff to understand the development of puppies in the population and whether any behaviour interventions are required. Together with these findings, this supports the usefulness of guide dog schools collecting behavioural information from volunteers that raise puppies within their programmes. This study found the r-PWQ to be predictive of guide dog success in an independent replication study on trainee guide dogs aged eight months. Furthermore, the r-PWQ has been able to distinguish between dogs that were successful or unsuccessful in guide dog training for juvenile guide dog puppies aged eight months when compared to a commonly used questionnaire, the C-BARQ. Based on the current findings, results suggest the r-PWQ could provide a better indication of future success as a guide dog for Guide Dogs UK at eight months of age than the C-BARQ. The r-PWQ trait scores were significantly linked to outcome in five out of nine trait scores, compared to two C-BARQ trait scores. Studies have previously reported that C-BARQ trait scores can be used to predict guide dog success [9] and C-BARQ trait scores for service dogs were significantly worse in dogs withdrawn for selected traits; Stranger-directed aggression, Stranger-directed fear, Non-social fear, Attachment and attention-seeking, Excitability and Energy level [11]. In contrast, the current study found the predictive ability of the C-BARQ to be low and there was no significant link between outcome as a guide dog and the C-BARQ traits of Non-social fear, Chasing, Trainability, Touch sensitivity, Attachment and attention-seeking and Energy level. Foyer et al. [19] similarly reported a low correlation between C-BARQ trait scores and success for German Shepherd military dogs at 14 months of age, supporting the need to review questionnaire predictive abilities between different working dog populations and to consider the age at completion. The difference in predictive ability between populations and cohorts may be due to the differing criteria for qualification, variation in training methods and age differences at questionnaire completion. Puppies in this study were aged eight months compared to other studies where the C-BARQ has been completed for puppies at varying ages, including, six, 12 and 14 months of age [9,11,19]. One question in the C-BARQ Chasing scale was recorded using a frequency scale in error, rather than severity scale, which may have impacted the results. However, the 0–4 scale still remained, and the trait was averaged from four questions, so the question did not have a large weighting to the overall score (in comparison the r-PWQ Animal Chase score that is formed of two questions scored on a frequency scale). The study could have further been improved if both sex and breed were controlled for in Z-scores. Z-scores were chosen for analyses as they are used in practice by Guide Dogs UK, however due to the sample size for some less well represented breeds, it was not possible to calculate reliable Z-scores controlling for both breed and sex in fear/anxiety traits.

The PWQ was initially designed, and the r-PWQ refined, to specifically fit this guide dog population. Furthermore, not all C-BARQ traits were included here as only traits comparable to the r-PWQ were analysed. Although many of the mean scores for C-BARQ and r-PWQ traits (see Table S3) between Guiding Eyes USA and Guide Dogs UK populations were similar, there did appear to be differences in Body Sensitivity and Attachment and attention seeking. Difference in practices between the two guide dog schools may account for the variation and further support the need for regular monitoring between and within populations. There were also some differences between the predictive abilities of the original PWQ when tested on the population it was developed with (see Table S2) and the current r-PWQ cohort. The order of questions in the r-PWQ and PWQ differed, and the questionnaire was completed on separate cohorts of dogs that may be impacted by uncontrolled for cohort effects, which could explain the results reported. Due to active breeding selection within working dog programmes a behavioural shift in the population may occur. It is suggested that the predictive ability of the questionnaire be reviewed periodically. Further studies could review the predictive ability of the C-BARQ compared to the r-PWQ at 12 months of age.

A commonly used measure of internal consistency for questionnaires is the Cronbach’s alpha, with scores of greater than 0.70 considered acceptable, although with a small number of items a lower figure can be accepted [16,23]. A previous study reported the Cronbach’s alpha of the PWQ scales Body sensitivity, Distractibility and General anxiety to be over 0.70 (0.73, 0.76 and 0.75 respectively) [8], similar to findings within this study. Many studies have also reported the internal consistency of the C-BARQ, with scores dependent on the population. C-BARQ traits were reported have Cronbach’s alpha values ranging from 0.39 for Energy Level to 0.92 for Stranger directed aggression in a population of Mexico pet dogs [20]. In comparison, a study on military dogs reported the highest Cronbach’s alpha to be 0.30 [19]. This study found five of the eight C-BARQ traits to have alpha values above 0.70. Three trait scores were found to have a Cronbach’s alpha of less than 0.70 for both the C-BARQ and r-PWQ (Separation-related, Trainability and Attachment and attention seeking). A reason for this may be the lower age of completion, as the C-BARQ is commonly reported to be completed between 12 and 14 months of age [5,15,19]. However, as trait scores from both the C-BARQ and r-PWQ produced the same level of inconsistency in responses, this may suggest the variation could be due to factors being assessed, or age of the dogs, rather than questionnaire design.

During the comparison there were more questions with missing data for C-BARQ which suggests that some questions may not be so relevant for the UK guide dog population. Extreme aggression behaviours are infrequently observed in guide dogs [14,15]. Unpublished data from this study found aggression related C-BARQ traits to be heavily “0” inflated (meaning that most scores were “0”) and therefore suggest these questions to not be appropriate for this guide dog population. These questions were omitted from the PWQ during development for this reason; where they do occur, they are captured by the standard training review process. Guide dog organisations can consider behaviours to be undesirable for a guide dog that may not be so in a pet dog. For instance, high levels of distraction are particularly important due to the unsuitability of a guide dog to be distracted when guiding a person with vision impairment. Similarly issues with body sensitivity can affect whether a dog is able to accept the contact and equipment essential to the guiding role. Therefore, a questionnaire for pet dogs may not be the most appropriate questionnaire to capture data on guide dogs.

It is important that guide dog organisations receive regular and completed behaviour reports for puppies to monitor progress. When determining the most appropriate behaviour questionnaire to implement in a programme, many factors require consideration, such as questionnaire length and scale. This study included the C-BARQ 100 because the questionnaire is used operationally by other guide dog schools and therefore could be compared to the r-PWQ. The r-PWQ is the shorter of the two questionnaires at 39 questions compared to 100 in the C-BARQ and so the demands on volunteer time are less which may result in higher participation and completion rates. A previous study reduced the length of the C-BARQ in attempts to minimise dropout rates [24], suggesting shorter questionnaires improve participation. Another difference between the two questionnaires is the scale used; the C-BARQ uses a five-point Likert scale, whereas the PWQ and r-PWQ use a visual analogue scale bar from 0–100, which has been reported to detect smaller changes when recording measures of behaviour [4]. However, where guide dog schools continue using different measures, this study has shown the ability for the two questionnaires to be compared by converting C-BARQ trait scores to a C-BARQ 100-point scale. There were good correlations between the r-PWQ and comparable C-BARQ traits, indicating that results could be considered somewhat analogous when comparing scores for dogs between different guide dog schools using different questionnaires. The r-PWQ could be further adapted to cover a wider range of behaviour traits and future work could include applications in other working/assistance dog populations, to inform breeding programmes and use of the questionnaire beyond prediction e.g., supporting behavioural interventions to improve success.

## 5. Conclusions

This study has reported the predictive validity of a puppy behaviour questionnaire for juvenile guide dogs (the r-PWQ), which was completed by the volunteers raising the puppies. When comparing the questionnaire to a commonly used behaviour questionnaire (C-BARQ), results suggest that the r-PWQ is more suitable and predictive for the UK guide dog population. Furthermore, the inclusion of a Distractibility trait, is a considerable advantage of the r-PWQ, as distraction is a common withdrawal reason for many guide dog schools and is not measured in the C-BARQ. For analogous C-BARQ and r-PWQ trait scores with a strong correlation (over *rho* of 0.60, such as Excitability, Attachment and attention-seeking, Chasing/Animal chase and Non-social fear/General anxiety), the results could be compared and used to review the behaviour of dogs moved between international guide dog schools.

## Figures and Tables

**Table 1:** Question group and wording for r-PWQ questionnaire, including the direction of association between the individual item within the group (negatively valanced items were transformed before average trait scores were calculated) and whether questions originated from the C-BARQ. Superscripts are used to indicate the origin of individual questions.

Group	Wording	Direction within Group	C-BARQ Originated?
r-Separation-Related Behaviour	Appears restless/agitated or paces when left, or about to be left ^2^	+	✓
	Whines when left, or about to be left ^2^	+	✓
	Barks when left, or about to be left ^2^	+	✓
	Chews/scratches at doors, floor, windows, curtains etc. when left, or about to be left ^2^	+	✓
	Loses its appetite when left, or about to be left ^2^	+	✓
	Appears agitated (whines, barks, howls, scratches at door etc.) when separated from you (or a member of the household) but not alone ^3^	+	X
r-Excitability	Exhibits a high degree of excitement (jumps up; barks; coughs etc.) just before being taken for a walk ^2,3^	+	✓
	Exhibits a high degree of excitement (jumps up; barks; coughs etc.) just before being taken on a car trip ^2,3^	+	✓
	Exhibits a high degree of excitement (jumps up; barks; coughs etc.) when visitors arrive at your home ^2,3^	+	✓
	Is excessive, difficult to control and if it lunges is hard to hold back	+	X
	Is hyperactive, restless, has trouble settling down ^2^	+	✓
	Barks persistently when alarmed or excited ^2^	+	✓
	Is self-controlled and calm [*appears also in Trainability*]	−	X
r-Attachment and attention seeking	Tends to follow you (or other member of household) about the house from room to room ^2^	+	✓
	Tends to nudge, nuzzle, or paw you (or others) for attention when you are sitting down ^2^	+	✓
	Becomes agitated (whines, jumps up, tries to intervene) when you (or others) show affection for another person ^2^	+	✓
	Becomes agitated (whines, jumps up, tries to intervene) when you show affection for another dog or animal ^2^	+	✓
	Displays a strong attachment for one particular member of the household ^2^	+	✓
	Returns directly to you if startled or frightened	+	X
Energy Level	Is active and energetic ^1^	+	✓
	Is playful ^1,3^	+	✓
General Anxiety	Is obviously disturbed by loud or unexpected sounds ^1,3^	+	✓
	Is spooked by odd or unexpected things or objects ^1,3^	+	✓
	Is anxious or uneasy in new situations ^1,3^	+	✓
	Backs away from or is reluctant to pass objects on the street (such as collecting boxes, bin bags or children’s ride-on toys)	+	X
Animal Chase ^	Chases birds or squirrels (or would like to) ^2^	+	✓
	Chases cats (or would like to) ^2^	+	✓
Body Sensitivity	Is uneasy with being physically handled/groomed	+	X
	Attempts to move away when you start to groom it	+	X
Distractibility	Pulls (including lunging) towards unfamiliar dogs	+	X
	Is motivated towards/distracted by food on the ground and or on tables/shelves	+	X
	Shows interest (attempts to greet, sniffs, wags tail) when passing children or members of the public	+	X
	Shows interest (attempts to greet, sniffs, wags tail) when encounters other dogs	+	X
Trainability	Attention can be attracted easily but it loses interest soon	−	X
	Will look at you when you talk to it directly in the home environment	+	X
	Attention can be easily distracted	−	X
	Is self-controlled and calm [*appears also in Excitability*]	+	X
	Needs obedience commands repeating to get a response	−	X
	Seems like it doesn’t listen even if it knows someone is speaking to it	−	X
	Stay’s/Wait’s when instructed to	+	X

✓ indicates the question originates from C-BARQ, more specifically from ^1^ Serpell & Hsu (2001); ^2^ Hsu & Serpell (2003); whilst ^3^ indicates the item wording was altered from the original C-BARQ form following Guide Dogs stakeholder feedback. ^ The two questions comprising the Animal Chase scale were added into the r-PWQ to address behaviour around animals.

**Table 2:** C-BARQ and r-PWQ traits that were directly compared throughout the study.

C-BARQ Trait	r-PWQ Trait
Separation-related behaviour	r-Separation-related behaviour *
Excitability	r-Excitability *
Non-social fear	General Anxiety
Chasing	Animal Chase *
Trainability	Trainability
Touch sensitivity	Body Sensitivity
Attachment and attention-seeking	r-Attachment and attention seeking *
Energy level	Energy *

* indicates an r-PWQ trait that included questions that originated from the corresponding C-BARQ scale.

**Table 3:** Cronbach’s alpha values for C-BARQ and r-PWQ traits.

C-BARQ Trait	Cronbach’s Alpha	r-PWQ Trait	Cronbach’s Alpha
Separation-related behaviour	0.56	r-Separation-related behaviour	0.60
Excitability	0.83	r-Excitability	0.79
Non-social fear	0.83	General Anxiety	0.79
Chasing	0.78	Animal Chase	0.74
Trainability	0.51	Trainability	0.59
Touch sensitivity	0.79	Body Sensitivity	0.79
Attachment and attention-seeking	0.66	r-Attachment and attention seeking	0.66
Energy level	0.77	Energy	0.88
		Distractibility	0.81

**Table 4:** Effect of breed and sex on C-BARQ and r-PWQ traits and whether breed was controlled for in trait scores.

Trait Group	Effect of Breed	Effect of Sex	Breed Controlled for?
C-BARQ Separation-related behaviour	r^2^ = 0.019, *p =* 0.531	r^2^ = 0.019, *p =* 0.157	
C-BARQ Excitability	r^2^ = 0.016, *p =* 0.472	r^2^ = 0.016, *p =* 0.305	
C-BARQ Non-social fear	r^2^ = 0.066, *p =* 0.007	r^2^ = 0.066, *p =* 0.025	✓
C-BARQ Chasing	r^2^ = 0.055, *p =* 0.020	r^2^ = 0.055, *p =* 0.106	✓
C-BARQ Trainability	r^2^ = 0.024, *p =* 0.128	r^2^ = 0.024, *p =* 0.873	
C-BARQ Touch sensitivity	r^2^ = 0.029, *p =* 0.091	r^2^ = 0.029, *p =* 0.454	
C-BARQ Attachment and attention-seeking	r^2^ = 0.012, *p =* 0.553	r^2^ = 0.012, *p =* 0.873	
C-BARQ Energy level	r^2^ = 0.015, *p =* 0.522	r^2^ = 0.015, *p =* 0.206	
r-PWQ Separation-related behaviour	r^2^ = 0.039, *p =* 0.030	r^2^ = 0.039, *p =* 0.240	✓
r-PWQ Excitability	r^2^ = 0.014, *p =* 0.451	r^2^ = 0.014, *p =* 0.539	
r-PWQ General Anxiety	r^2^ =0.063, *p =* 0.002	r^2^ = 0.063, *p =* 0.036	✓
r-PWQ Animal Chase	r^2^ = 0.054, *p =* 0.004	r^2^ = 0.054, *p =* 0.087	✓
r-PWQ Trainability	r^2^ = 0.033, *p =* 0.071	r^2^ = 0.033, *p =* 0.180	
r-PWQ Body Sensitivity	r^2^ = 0.016, *p =* 0.469	r^2^ = 0.016, *p =* 0.347	
r-PWQ Attachment and attention seeking	r^2^ = 0.012, *p =* 0.799	r^2^ = 0.012, *p =* 0.143	
r-PWQ Energy	r^2^ = 0.007, *p =* 0.963	r^2^ = 0.007, *p =* 0.200	
r-PWQ Distractibility	r^2^ = 0.011, *p =* 0.567	r^2^ = 0.011, *p =* 0.868	

✓ indicates the factor was controlled for in trait scores.

**Table 5:** Correlation coefficient (Spearman’s rank) between Z-scores for C-BARQ and r-PWQ traits.

C-BARQ Trait	r-PWQ Trait	Spearman’s *rho*	*p* Value
Separation-related behaviour	r-Separation-related behaviour	−0.07	0.193
Excitability	r-Excitability	0.66	<0.001
Non-social fear	General Anxiety	0.71	<0.001
Chasing	Animal Chase	0.78	<0.001
Trainability	Trainability	0.43	<0.001
Touch sensitivity	Body Sensitivity	0.42	<0.001
Attachment and attention-seeking	r-Attachment and attention seeking	0.70	<0.001
Energy level	Energy	0.48	<0.001

**Table 6:** The Wald Chi-square, odds ratio (OR), confidence intervals (CI) and the significance value for C-BARQ and PBQ trait scores as predictors for qualification as a guide dog.

Trait Group	C-BARQ or r-PWQ	Wald	*p*	OR	95% CI	Predictive?
Separation-related	C-BARQ	5.716	0.017	0.298	(0.104, 0.799)	✓
	r-PWQ	0.057	0.811	1.032	(0.798, 1.333)	
Excitability	C-BARQ	3.884	0.049	0.701	(0.492, 0.998)	✓
	r-PWQ	10.903	0.001	0.649	(0.502, 0.839)	✓
Fear/anxiety	C-BARQ	2.773	0.096	0.810	(0.632, 1.038)	
	r-PWQ	1.249	0.264	0.875	(0.692, 1.106)	
Chasing/Animal Chase	C-BARQ	0.276	0.600	0.924	(0.687, 1.242)	
	r-PWQ	4.313	0.038	0.770	(0.601, 0.985)	✓
Trainability	C-BARQ	1.706	0.191	1.190	(0.916, 1.546)	
	r-PWQ	5.4388	0.020	1.364	(1.049, 1.772)	✓
Sensitivity	C-BARQ	2.703	0.100	0.775	(0.572, 1.050)	
	r-PWQ	1.750	0.186	0.841	(0.651, 1.087)	
Attachment and attention seeking	C-BARQ	1.113	0.291	0.867	(0.666, 1.130)	
	r-PWQ	4.409	0.036	0.762	(0.592, 0.982)	✓
Energy	C-BARQ	2.272	0.132	0.819	(0.632, 1.062)	
	r-PWQ	2.689	0.101	0.800	(0.613, 1.045)	
Distractibility	r-PWQ	16.230	<0.001	0.571	(0.434, 0.750)	✓

✓ indicates trait scores is significantly linked (*p* < 0.05) to guide dog success.

## Supplementary Materials

The following are available online at https://www.mdpi.com/2076-2615/10/12/2382/s1, Table S1: Sample sizes for each of the three assessments as denoted by age of the dog at the time of assessment, Table S2: Each of the 61 items from the original PWQ shown with P-values for predictive validity from logistic regression models of each individual question, at each sampled age, against training outcome (qualified or withdrawn for behaviour) for the original cohort of dogs. Associations that met each steps criterion for retention in the r-PWQ are highlighted in bold. Step 2 analyses were only conducted for individual items that failed to meet Step 1 criteria, Table S3: Mean (±S.D.) trait scores, Mann-Whitney U and significance values for Guide Dogs UK and Guiding Eyes populations for C-BARQ and r-PWQ comparable traits (with the addition of the r-PWQ Distractibility).
