# Supplementary material for: Concurrent and Predictive Criterion Validity of a Puppy Behaviour Questionnaire for Predicting Training Outcome in Juvenile Guide Dogs
Source: Animals (Basel). 2020 Dec 11;10(12):2382. doi: 10.3390/ani10122382 (PMC7763496; doi:10.3390/ani10122382)
Supplement: Supplementary file 1 [file animals-10-02382-s001.pdf]

animals

MDPI

Article

Concurrent and Predictive Criterion Validity of a Puppy Behaviour Questionnaire for Predicting Training Outcome in Juvenile Guide Dogs

Rebecca L. Hunt [1,*], Gary C.W. England [2], Lucy Asher [3], Helen Whiteside [1] and Naomi D. Harvey [2]

[1] Guide Dogs National Breeding Centre, Banbury Road, Bishops Tachbrook, Warwickshire CV33 9WF, UK; Helen.VaterlawsWhiteside@guidedogs.org.uk
[2] School of Veterinary Science & Medicine, University of Nottingham, Sutton Bonington Campus, Leicestershire LE12 5RD, UK; gary.england@nottingham.ac.uk (G.C.W.E.), naomi.harvey@nottingham.ac.uk (N.D.H.)
[3] Centre for Behaviour and Evolution, School of Natural and Environmental Sciences, Newcastle University, Agriculture Building, Newcastle NE1 7RU, UK; lucy.asher@newcastle.ac.uk
* Correspondence: Becky.Hunt@guidedogs.org.uk

1. Refining the PWQ to create the r-PWQ

1.1. Materials and Methods

Here, we detail the methods used to refine the original Puppy Walker Questionnaire (PWQ) into a shorter version for applied use within Guide Dogs, UK. Development of the original PWQ is described in full in [1]. In brief, the questionnaire was developed based upon previously published literature in consultation with Guide Dogs staff and puppy walkers, to capture behavioural scores for puppies in their first year of life that would be relevant to their personality and likely suitability as guide dogs. Following development, the final PWQ questionnaire contained 61 items asking puppy walkers to rate their dog's behaviour over the past month on a 100mm visual analogue scale with the anchors "Never" and "Almost Always" (original questions are listed alongside the results in Table S2). Of the 61 items, 20 were from the C-BARQ and were retained in the same scales with the same names; Excitability, Separation-related behaviour and Attachment and attention-seeking. The remaining questions split via reliability analysis into a miscellaneous group and five scales with acceptable internal consistency, inter-rater reliability and test-retest reliability over the three ages named: Trainability; Body Sensitivity; Distractibility; General Anxiety; Stair Anxiety and Energy.

In order for the PWQ to be of most practical use to Guide Dogs, the questionnaire was refined to reduce the number of questions asked to contain only those with potential for predictive associations with qualification/withdrawal, plus scales which demonstrate temporal consistency (an indicator of personality) but lacked predictive associations, for use in profiling.

1.2. Participants

The same data were used to refine the PWQ as were used to develop it originally. Guide Dogs puppy walkers (PWs) of dogs that turned five months of age between October and December 2012 (n = 311) were invited to complete the PWQ at three points during the first year of the dog's life; when dogs were aged five, eight and twelve months. After initially opting into the study, invitations to complete the PWQ were sent by post or email (at the puppy walkers request) two weeks prior to the date the dogs were due to turn five, eight and twelve months of age. Puppy walkers of dogs that participated in a behavioural test at the same three age points were also invited to complete the PWQ. In total, 276 dogs (130M/146F) had at least one completed PWQ. The dogs comprised eight breeds or crossbreeds (Golden retriever $_{Sire}$ × Labrador $_{Dam}$, 105; Labrador, 65; Golden retriever, 30; Labrador $_{Sire}$ × Golden retriever crossbreed $_{Dam}$, 29; Golden retriever × German Shepherd Dog, 24; German Shepherd Dog, 16; Labrador $_{Sire}$ × Golden retriever $_{Dam}$, 5; Labrador × Labrador crossbreed, 2). The dogs were a mean age of 5.17 months (S.D. ± 8 days) for the first assessment, 8.17 months (± 7 days

S.D.) for the second assessment and 12.04 months (± 12 days S.D.). For the purpose of this study, only dogs that had qualified as a guide dog or been withdrawn permanently for behavioural reasons were included in this analysis (Table S1). Dogs the entered the breeding program (n = 14), were withdrawn for health reasons (n = 16), transferred to other organisations (n = 3) or deceased (n = 1) were all excluded from this analysis.

Table S1. Sample sizes for each of the three assessments as denoted by age of the dog at the time of assessment.

Age (months)	Total	Withdrawn	Qualified
5	263	72	157
8	214	51	129
12	226	53	139

1.3. Refining the PWQ

In the original published PWQ [1], scales that came from the Canine Behavioral Assessment and Research Questionnaire (C-BARQ) were presented in the form they take in the C-BARQ for the purposes of comparability of results. However, for operational purposes within Guide Dogs permission was granted by the C-BARQ creator, Prof James Serpell, to change the composition of the scales to optimise performance for applied use. For this purpose, a new internal reliability analysis was performed using the C-BARQ items for the scale Excitability and six of the miscellaneous items that were hypothesised to also be measuring behaviour related to excitability. These six items ("Is hyperactive, restless, has trouble settling down"; "Is self-controlled and calm"(negatively transformed); "Barks persistently when alarmed or excited"; "Is calm and quiet"(negatively transformed); "Is excessive and if it lunges is hard to hold back" and "Jumps up on people (stands to place front paws on persons/chest/legs)") were successfully grouped with the C-BARQ items achieving a high Cronbach's alpha value of 0.85 (using the scores for 12 month old dogs), suggesting they could be reliably averaged to create a new scale score for Excitability. The miscellaneous question "Returns directly to you if startled or frightened" was designed to evaluate the secure base aspect of attachment [3], so we also tested the internal reliability of the C-BARQ Attachment and attention-seeking scale if this was included in it. The Cronbach's alpha score did not differ significantly when this question was added (0.54 originally reduced slightly to 0.53) so this question was retained as part of the Attachment and attention-seeking scale prior to predictive refinement. Following the alterations to the two described scales, individual items from each PWQ scale were examined for predictive associations in order to identify questions that could be removed.

To reduce the length of the questionnaire, none of the remaining miscellaneous questions were included in the r-PWQ, as averaged scale scores were considered to be more useful for Guide Dogs. Two steps of analysis were used to identify individual items from within the scales to retain for future use. In Step 1, all individual items were evaluated for potential associations with qualification or withdrawal for behaviour using univariate logistic regressions models. All items that showed an association with qualification or withdrawal to a 90% confidence level ($p < 0.1$) for at least one of the three ages were retained for use in the r-PWQ as being potentially predictive. Where a scale contained some items that were associated with outcome (for at least one age) and some that were not associated at any age, all of those items with no predictive association were removed, so that future scale-average scores would be made using only items with potential predictive value. If a whole scale did not contain any items with potential predictive value, the items were checked for inter-rater reliability and temporal consistency as part of Step 2, and the scale was kept only if both of these criteria were met for all items.

To test for predictive associations, each individual item from the PWQ was assessed for potential associations with qualification or withdrawal for behaviour using univariate logistic regressions models. Separate analyses were conducted for each item for the five, eight and twelve-month PWQs. The basic model equation using a logit link function can be written as:

$$y_i \sim \text{Binomial}(n_i, \pi_i) \quad (1)$$

$$\text{logit}(\pi_i) = \log = \beta_0 + \beta_1 X_i \quad (2)$$

Where y_i represents the response variable (withdrawal for behaviour vs. entry in to advanced training or qualified) for the ith dog; π_i represents the probability that $y_i = 1$; β_0 is the model intercept (the estimated response value when the predictor equals zero), and the regression coefficient for the explanatory variable is represented by $\beta_1 X_i$.

This analysis provided statistics representing predictive associations for each individual item as scored at each of the three ages. All items which showed an association with qualification or withdrawal to a 90% confidence level ($p < 0.1$) were retained for further analysis. Retained items were kept in their original PWQ groups, even if some of the group's items had been excluded. Trait scores were calculated as means of all items within the groups, with those worded negatively in relation to the rest of the scale changed in direction (100-item score).

To assess temporal consistency, correlation estimates were sought between the scores given to all dogs (n = 176) that had an assessment completed at each age (five, eight and twelve months). Two-way random intra-class correlation coefficients (ICC) with the consistency method were used to provide a coefficient that summed the overall consistency between the three assessments. Items which achieved ICC's of >0.30 (±0.01) were considered to show acceptable temporal consistency. Inter-rater reliability (evaluated using a 2-way mixed ICC model with a consistency method) was accepted for items statistically significant to $p < 0.05$ (n = 21 pairs of puppy walkers living with and scoring the same dog, methods for data collection described in full in [1]).

The inter-rater and temporal consistency analyses were only done for individual items not meeting the criteria for Step 1, for the purpose of refining the PWQ. Scale level temporal consistency and inter-rater reliability is described in full in [1] for the novel PWQ scales and for the C-BARQ scales has been described in other studies e.g. [2].

2. Results

In total, 39 items were included in the r-PWQ (Table S2). A new scale named Animal Chase was added to the r-PWQ, containing two questions originating from the C-BARQ scale Chasing ("Chases birds or squirrels (or would like to)" and "Chases cats (or would like to)") and three scales had some items removed due to lack of individual associations with training outcome, or lack of reliability. In the r-PWQ, these scales are referred to as r-Attachment and attention seeking (r-AAS), r-Separation-related behaviour (r-SRB) and r-Excitability to indicate that they have been refined as compared to their original form in the original PWQ.

Table S2. Each of the 61 items from the original PWQ shown with P-values for predictive validity from logistic regression models of each individual question, at each sampled age, against training outcome (qualified or withdrawn for behaviour) for the original cohort of dogs. Associations that met each steps criterion for retention in the r-PWQ are highlighted in bold. Step 2 analyses were only conducted for individual items that failed to meet Step 1 criteria.

Item	Step 1			Step 2		Decision
	Predictive Validity (*p*-Values) (n = 176)			Inter-rater Reliability (n = 21 pairs)	Temporal Consistency (n=176)	
	5M	8M	12M	ICC	ICC	
**Attachment and attention seeking**						
Tends to follow you (or other member of household) about the house from room to room [2]	0.308	0.634	0.558	**0.47 ***	**0.34 ***	Kept
Tends to nudge, nuzzle, or paw you (or others) for attention when you are sitting down [2]	0.109	0.192	0.486	**0.48 ***	**0.36 ***	Kept
Becomes agitated (whines, jumps up, tries to intervene) when you (or others) show affection for another person [2]	0.590	0.240	0.982	**0.37 ***	**0.32 ***	Kept
Becomes agitated (whines, jumps up, tries to intervene) when you show affection for another dog or animal[2]	0.173	0.632	0.845	**0.49 ***	**0.27 ***	Kept
Displays a strong attachment for one particular member of the household [2]	0.737	0.383	0.798	**0.61 ****	**0.33 ***	Kept
Returns directly to you if startled or frightened [6]	0.456	0.554	0.402	**0.36 ***	**0.27 ***	Kept
Tends to sit close to or in contact with you (or others) when you are sitting down [2]	0.323	0.548	0.758	−0.02		Rejected
**Separation-related behaviour**						
Appears restless/agitated or paces when left, or about to be left [2]	0.974	0.803	**0.010**			Kept
Whines when left, or about to be left [2]	**0.066**	0.738	**0.006**			Kept
Barks when left, or about to be left [2]	**0.053**	**0.038**	**0.003**			Kept
Chews/scratches at doors, floor, windows, curtains etc. when left, or about to be left [2]	**0.046**	**0.015**	0.349			Kept
Loses its appetite when left, or about to be left [2]	0.636	**0.023**	0.242			Kept
Appears agitated (whines, barks, howls, scratches at door etc.) when separated from you (or a member of the household) but not alone [6]	0.110	**0.056**	**0.003**			Kept
Shakes shivers of trembles when left, or about to be left [2]	0.265	0.172	0.345	−0.06		Rejected
Salivates excessively when left, or about to be left [2]	0.779	0.277	0.255	0.05		Rejected
Howls when left, or about to be left [2]	0.157	0.244	0.102	0.28		Rejected
**Excitability**						
Exhibits a high degree of excitement (jumps up; barks; coughs etc.) when playing with you or other members of the household [2,7]	0.438	0.140	0.441	**0.35 ***	**0.37 ***	Rejected
Exhibits a high degree of excitement (jumps up; barks; coughs etc.) just before being taken for a walk [2,7]	0.213	**0.016**	**0.019**			Kept
Exhibits a high degree of excitement (jumps up; barks; coughs etc.) just before being taken on a car trip [2,7]	0.303	0.109	**0.089**			Kept
Exhibits a high degree of excitement (jumps up; barks; coughs etc.) when visitors arrive at your home [2,7]	0.235	**0.074**	0.349			Kept

Exhibits a high degree of excitement (jumps up; barks; coughs etc.) when you or other members of the household come home after a brief absence [2, 7]	0.344	0.634	0.696	**0.41 ***	**0.31 ***	Rejected
Is hyperactive, restless, has trouble settling down [2]	**0.008**	**0.014**	**0.033**			Kept
Is self-controlled and calm [6; A]	**0.024**	**0.015**	**0.008**			Kept
Barks persistently when alarmed or excited [2]	**0.001**	**0.042**	**0.027**			Kept
Is calm and quiet [6]	0.510	0.271	0.314	**0.51 ****	**0.30 ***	Rejected
Is excessive, difficult to control and if it lunges is hard to hold back [4]	**0.079**	**0.065**	**0.079**			Kept
Jumps up on people (stands to place front paws on persons chest/legs) [6]	0.663	0.291	0.926	**0.56 ****	**0.49 ***	Rejected
**Energy**						
Is playful [1; 7; A]	0.725	0.944	0.258	**0.74 ****	**0.29 ***	Kept
Is active and energetic [1; A]	0.780	0.904	0.856	**0.77 ****	**0.29 ***	Kept
**General Anxiety**						
Is obviously disturbed by loud or unexpected sounds [1, 7]	**0.068**	**0.019**	0.148			Kept
Is obviously spooked by odd or unexpected things or objects [1, 7]	0.411	0.337	**0.022**			Kept
Is anxious or uneasy in new situations [1, 7]	0.860	**0.022**	0.214			Kept
Backs away from or is reluctant to pass objects on the street (such as collecting boxes, bin bags or children's ride-on toys) [5]	**0.066**	**0.088**	**0.026**			Kept
**Body Sensitivity**						
Is uneasy with being physically handled/groomed [6]	**0.077**	**0.072**	0.656			Kept
Attempts to move away when you start to groom it [6]	**0.042**	**0.053**	0.153			Kept
**Distractibility**						
Pulls (including lunging) towards unfamiliar dogs [6]	**0.017**	**0.084**	**0.074**			Kept
Is motivated towards/distracted by food on the ground and or tables/shelves [3, 6]	0.380	0.233	**0.097**			Kept
Shows interest (attempts to greet, sniffs, wags tail) when directly approached by children or member of the public [6]	0.123	0.302	0.302	0.10	0.23	Rejected
Shows interest (attempts to greet, sniffs, wags tail) when passing children or members of the public [6]	**0.077**	**0.072**	0.656			Kept
Shows interest (attempts to greet, sniffs, wags tail) when encounters other dogs [6]	**0.042**	**0.053**	0.153			Kept
**Trainability**						
Attention can be attracted easily but it loses interest soon [4; 7]	**0.039**	**0.186**	**0.017**			Kept
Will look at you when you talk to it directly in the home environment [6; 7]	0.411	**0.054**	0.898			Kept
Attention can be easily distracted [4]	**0.028**	**0.019**	**0.010**			Kept
Needs obedience commands repeating to get a response [5]	0.710	**0.030**	0.266			Kept
Seems like it doesn't listen even if it knows someone is speaking to it [4]	0.734	0.288	**0.007**			Kept
Stay's/Wait's when instructed to [6]	0.196	0.210	**0.037**			Kept
Is attentive to you [6; A]	0.582	0.710	0.644	0.10		Rejected
Learns new things quickly [6]	0.364	0.476	0.516	**0.58 ****	0.15	Rejected
Will respond immediately to the recall command while off lead [6]	0.656	0.472	0.125	**0.75 ****	0.10	Rejected
Is responsive to/focussed on you whilst playing retrieve games [6]	0.826	0.676	0.816	**0.75 ****	0.21	Rejected
Fidgets all the time [4]	0.332	0.870	0.129	**0.67 ****	0.23	Rejected

[*This question is also in Excitability. Statistics are shown there*] Is self-controlled and calm [6; A]		See above				Kept
**Stair anxiety**						
Appears uneasy on closed stairs [6;7]	0.459	0.287	0.910	0.05		Rejected
Appears uneasy on open or unusual stairs [6;7]	0.593	0.968	0.917	**0.52 ****	0.24	Rejected
**Miscellaneous**						
Attempts to steal food [6]	0.106	**0.028**	**0.007**			Rejected
Appears uneasy or uncomfortable when putting on Guide Dog equipment (including collars) [6]	0.627	0.803	**0.070**			Rejected
Likes to carry objects in their mouth [6]	0.830	**0.083**	0.249			Rejected
Plays by itself [6]	0.411	0.687	0.534	**0.45 ****	**0.60**	Rejected
Is the first to initiate play with you [6]	**0.089**	0.426	0.809			Rejected
When settled this dog reacts quickly to disturbances [6]	0.138	**0.053**	**0.075**			Rejected
Tucks tail under, flattens ears, whines or trembles when being physically handled/groomed [6]	0.718	0.583	0.131	0.09		Rejected
Is initially excitable (jumps up; barks; coughs etc), but quickly settles [6]	0.887	0.359	0.202	0.23		Rejected

Numbers in superscript represent the origin of the item: [1] Serpell & Hsu (2001); [2] Hsu & Serpell (2003); [3] Arata et al (2010); [4] Vas et al (2007); [5] Guide Dogs PW survey; [6] new items; [7] items that were altered or created following panel feedback. [A] indicates that the anchors for the 100mm VAS scale were "Really does not describe this dog" to "Really describes this dog", whilst all remaining items were scored on a frequency scale from "Never" to "Almost Always".

All of the miscellaneous questions were excluded from the r-PWQ, as averaged scale scores were considered to be more useful for Guide Dogs and this helped to shorten the length of the questionnaire. When individual items within trait scales were not predictive but others were, they were excluded from the r-PWQ even if they met the criterion for Step 2, in order to create scales that were averages only of questions with predictive potential. The two questions that comprise the scale Energy were not predictive, but met Step 2 criteria, so this scale was retained as it could still be used to form a scale useful for profiling purposes if not for prediction.

One question was removed from the C-BARQ-derived scale Attachment and attention-seeking, three were removed from the C-BARQ-derived scale Separation-related behaviour. Four questions were removed from the Excitability scale (two of these were original C-BARQ items and two were newly added ones) due to lacking predictive associations. In the r-PWQ, these scales will be referred to as r-AAS, r-SRB and r-Excitability to indicate that they have been refined as compared to their original form in the original PWQ and C-BARQ.

Two additional questions from the C-BARQ scale Chasing were added into the r-PWQ as Guide Dogs wanted to evaluate how dogs reacted to animals. The two questions were worded as follows "Chases birds or squirrels (or would like to)" and "Chases cats (or would like to)" and were averaged to make a C-BARQ-derived score called Animal Chase.

3. Comparison between Guide Dog populations

3.1. Materials and Methods

The mean and standard deviation (reported as ± S.D.) for r-PWQ and C-BARQ comparable traits, with the addition of the r-PWQ Distractibility trait were calculated from a population of 359 dogs (n=321 Guide Dogs UK, n=38 Guiding Eyes). Mann-Whitney U tests were used to compare scores between populations and results reported as significant when $P < 0.05$.

3.2. Results

Mean scores were similar between Guiding Eyes and Guide Dogs UK populations. Scores for Body Sensitivity and Attachment and attention seeking traits in both the r-PWQ and C-BARQ showed the greatest difference between populations (see Table S3).

Table S3. Mean (± S.D.) trait scores, Mann-Whitney U and significance values for Guide Dogs UK and Guiding Eyes populations for C-BARQ and r-PWQ comparable traits (with the addition of the r-PWQ Distractibility).

Trait Group	Guide Dogs UK mean (±S.D.)	Guiding Eyes mean (±S.D.)	Mann-Whitney U	Z	*p-Value*
C-BARQ Separation-related behavior	0.141 (0.237)	0.196 (0.278)	5364.000	−1.065	0.287
C-BARQ Excitability	1.510 (0.740)	1.359 (0.581)	5286.000	−1.263	0.206
C-BARQ Non-social fear	0.441 (0.515)	0.338 (0.377)	5258.000	−1.129	0.259
C-BARQ Chasing	1.264 (0.803)	1.035 (0.777)	3362.000	−1.577	0.115
C-BARQ Trainability	2.750 (0.371)	2.890 (0.366)	4925.000	−1.949	0.051
C-BARQ Touch sensitivity	0.167 (0.336)	0.574 (0.730)	3518.500	−4.617	<0.001 *
C-BARQ Attachment and attention-seeking	1.403 (0.598)	1.709 (0.534)	4024.500	−3.105	0.002 *
C-BARQ Energy level	2.592 (0.712)	2.566 (0.670)	5901.500	−0.336	0.737
r-PWQ Separation-related behaviour	4.396 (6.749)	4.947 (7.855)	5702.000	−0.542	0.588
r-PWQ Excitability	22.565 (16.199)	16.917 (12.723)	4381.000	−2.045	0.041 *
r-PWQ General Anxiety	9.010 (13.035)	4.842 (5.551)	5111.000	−1.584	0.113
r-PWQ Animal Chase	29.643 (26.054)	23.763 (21.795)	5422.000	−1.119	0.263
r-PWQ Trainability	71.458 (14.947)	75.154 (12.053)	5220.500	−1.398	0.162
r-PWQ Body Sensitivity	8.937 (14.690)	17.026 (22.964)	4720.000	−2.253	0.024 *
r-PWQ Attachment and attention seeking	30.568 (17.748)	35.725 (13.312)	4713.000	−2.031	0.042 *
r-PWQ Energy	77.026 (20.849)	70.829 (18.685)	4747.000	−2.236	0.025 *

r-PWQ Distractibility	44.914 (24.182)	37.914 (20.095)	5000.500	−1.816	0.069

* indicates *p*-values < 0.05.

References

1. Harvey, N. D.; Craigon, P. J.; Blythe, S. A.; England, G. C. W.; Asher, L. Social rearing environment influences dog behavioral development. *J. Vet. Behav. Clin. Appl. Res.* **2016**, *16*, 13–21, doi:10.1016/j.jveb.2016.03.004.
2. Duffy, D. L.; Serpell, J. A. Predictive validity of a method for evaluating temperament in young guide and service dogs. *Appl. Anim. Behav. Sci.* **2012**, *138*, 99–109, doi:10.1016/j.applanim.2012.02.011.
3. Wanser, S.H.; Udell, M.A.R. Does attachment scurity to a human handler influence the behavior of dogs who engage in animal assisted activities. *Appl. Anim. Behav. Sci.* **2019,** *210*, 88-94, doi:10.1016/j.applanim.2018.09.005.
4. Serpell, J.A.; Hsu, Y.Y. Development and validation of a novel method for evaluating behavior and temperament in guide dogs. *Appl. Anim. Behav. Sci.*, **2001**, *72*, 347-364, doi: 10.1016/S0168-1591(00)00210-0.
5. Hsu, Y.Y; Serpell, J.A. Development and validation of a questionnaire for measuring behavior and temperament traits in pet dogs. *J. Am. Vet. Med. Assoc*. **2003**, *223,* 1293-1300, doi:10.2460/javma.2003.223.1293
6. Arata, S; Momozawa, Y.; Takeuchi, Y.; Mori, Y. Important behavioral traits for predicting guide dog qualification. *J. Vet. Med. Sci.* **2010**, *72*, 539-545, doi: 10.1292/jvms.09-0512.
7. Vas, J., Topál, J.; Péch É.; Miklósi, Á. Measuring attention deficit and activity in dogs: A new application and validation of a human ADHD questionnaire. *Appl. Anim. Behav. Sci.* **2007**, *103*, 105-117, doi: 10.1016/j.applanim.2006.03.017.

Publisher's Note: MDPI stays neutral with regard to jurisdictional claims in published maps and institutional affiliations.

© 2020 by the authors. Licensee MDPI, Basel, Switzerland. This article is an open access article distributed under the terms and conditions of the Creative Commons Attribution (CC BY) license (http://creativecommons.org/licenses/by/4.0/).
